# Selective Neural Entrainment Reveals Hierarchical Tuning to Linguistic Regularities in Reading

**DOI:** 10.1162/nol_a_00145

**Published:** 2024-06-03

**Authors:** Mara De Rosa, Lorenzo Vignali, Anna D’Urso, Maria Ktori, Roberto Bottini, Davide Crepaldi

**Affiliations:** Cognitive Neuroscience Department, International School for Advanced Studies, Trieste, Italy; Center for Mind/Brain Sciences (CIMeC), University of Trento, Mattarello, Trento, Italy

**Keywords:** identification, linguistic units, neural entrainment, reading, statistical learning, visual word

## Abstract

Reading is both a visual and a linguistic task, and as such it relies on both general-purpose, visual mechanisms and more abstract, meaning-oriented processes. Disentangling the roles of these resources is of paramount importance in reading research. The present study capitalizes on the coupling of fast periodic visual stimulation and MEG recordings to address this issue and investigate the role of different kinds of visual and linguistic units in the visual word identification system. We compared strings of pseudo-characters; strings of consonants (e.g., sfcl); readable, but unattested strings (e.g., amsi); frequent, but non-meaningful chunks (e.g., idge); suffixes (e.g., ment); and words (e.g., vibe); and looked for discrimination responses with a particular focus on the ventral, occipito-temporal regions. The results revealed sensitivity to alphabetic, readable, familiar, and lexical stimuli. Interestingly, there was no discrimination between suffixes and equally frequent, but meaningless endings, thus highlighting a lack of sensitivity to semantics. Taken together, the data suggest that the visual word identification system, at least in its early processing stages, is particularly tuned to form-based regularities, most likely reflecting its reliance on general-purpose, statistical learning mechanisms that are a core feature of the visual system as implemented in the ventral stream.

## INTRODUCTION

In today’s complex societies, printed words are among the most culturally relevant visual objects processed by the human brain. Visual word recognition is dexterously mastered by skilled readers, who can process written input as swiftly as they do animals, plants, or faces. However, printed words are not natural objects: reading and writing constitute a relatively recent invention (Carr, 1999), making the literate brain a case of an expert system shaped by cultural experience (Dehaene & Cohen, 2011). As such, the neural underpinnings of reading have attracted much attention as a window not only into the computational structure of the brain, but also into learning and plasticity (e.g., Carreiras et al., 2014; van Paridon et al., 2021).

Consistent evidence suggests the literate system relies on cortical resources originally deputed to other functions (e.g., visual object processing) and reorganized (or even recycled, Dehaene & Cohen, 2007) during literacy acquisition (as reviewed in, e.g., Dehaene et al., 2015). As a result, the neural underpinnings of reading would still follow the principles that govern object recognition in the ventral stream (Dehaene et al., 2005). A clear example comes from the hierarchical organization observed in the macaque monkey, with an anterior-to-posterior progression of increasingly larger receptor fields for more complex objects (Booth & Rolls, 1998; Riesenhuber & Poggio, 1999; Rolls, 2001). This structure might be ideally suited for language; many theories, particularly of visual word identification, stipulate a hierarchy of progressively larger processing units, from letters, to morphemes, to words (e.g., Crepaldi et al., 2010; Grainger & Beyersmann, 2017; Grainger & Ziegler, 2011; Taft & Nguyen-Hoan, 2010).

Evidence for a graded selectivity for increasingly complex linguistic stimuli was observed by Vinckier et al. (2007) in a portion of the ventral occipitotemporal cortex (vOT), with the asymmetrical left profile that is generally associated with linguistic processing (Cohen et al., 2002; Dejerine, 1892; Warrington & Shallice, 1980). Vinckier et al. (2007) exposed skilled readers to a hierarchy of linguistic stimuli, ranging from strings of pseudo-characters to real words, and including sequences of infrequent letters, frequent letters but rare bigrams, and frequent bigrams but rare quadrigrams. Blood oxygen level dependent (BOLD) contrast responses indicated the presence of a hierarchical trajectory, with progressively selective responses for more complex stimuli. Moreover, this hierarchy unfolded in a posterior-to-anterior gradient, again in line with a fundamental organizational principle of the visual identification system more generally (e.g., DiCarlo et al., 2012).

In addition to unveiling the general organization of the orthographic system in the ventral stream, Vinckier et al.’s (2007) data raise several interesting questions. First, the hierarchy of stimuli that they used is entirely based on visual familiarity (i.e., frequency). Frequency of occurrence is a crucial element in the visual word identification system (e.g., Coltheart et al., 2001; Forster & Chambers, 1973; Norris, 2006). However, there are also units that, on top of frequency, have a peculiar linguistic status that would place them somewhat higher in this hierarchy (e.g., graphemes, such as “ch” in “chart,” or morphemes, such as “er” in “dealer,” might be more relevant for skilled readers compared to frequent, but linguistically unmarked units, e.g., “el” in “chapel”). Indeed, such higher linguistic constructs were repeatedly shown to have a crucial functional role in word identification (e.g., Amenta & Crepaldi, 2012; Grainger et al., 1991); what is then the relationship between said units, and between the letter chunks considered in Vinckier et al. (2007)?

Second, Vinckier et al. (2007) found that real words did not differ from highly frequent, ortho-phonotactically legal quadrigrams (i.e., pseudowords). Albeit in line with previous evidence (Binder et al., 2006; Dehaene et al., 2005; Pammer et al., 2004; Price et al., 1996; Wydell et al., 2003), the lack of a reliable neural distinction between words and pseudowords has been the object of a heated debate (as detailed in, e.g., Price, 2012; Taylor et al., 2013). Indiscriminate responses to real words (e.g., “rent”) and well-structured letter strings, previously unseen and meaningless (e.g., “tren”), suggest that visual word recognition might be entirely supported by abstract orthographic knowledge, consolidated by frequent encounters with regularly co-occurring patterns and beyond any lexical information. In line with this observation, some models of visual word identification (Baayen et al., 2011; Binder et al., 2006; Xu & Taft, 2014) have entirely disposed of an orthographic lexicon—a functional and neural repository that stores word representations—which has been at the core of neuropsychological models of reading in the 20 th century (e.g., Morton, 1969; Patterson et al., 1987; Wernicke, 1874). These sublexical accounts have been challenged on the basis of studies showing selective lexical responses (Glezer et al., 2009; Kronbichler et al., 2007; Kronbichler et al., 2009), and proposing an interactive account of linguistic information, which would provide a source of top-down feedback for the early processing of orthographic input (e.g., Carreiras et al., 2014; Heilbron et al., 2020; Price & Devlin, 2003, 2011). In this breed of theories, both orthographic features and higher-order information (like phonology or semantics), as well as their mutual interaction, are at the core of the neural computations performed in the literate brain. A strict integration of information across the linguistic system has been also pushed by data showing early phonological and semantic effects upon the presentation of written words (e.g., Amenta et al., 2015; Chen et al., 2015; Rastle & Brysbaert, 2006; Sulpizio et al., 2022). Findings are still somewhat inconsistent on this topic, particularly on the neuroimaging side (e.g., Vignali et al., 2023, could not find reliable semantic effects until 320 ms after stimulus onset, well after the emergence of orthographic effects). Overall, however, they certainly yield some credibility to the hypothesis that orthographic information might be strongly coupled with phonology and semantics, perhaps to the point that there is no, or very little temporal separation between. Overall, this ongoing debate clearly indicates that while we might have achieved rather refined knowledge of *where* the neural underpinnings of reading reside, we still have many important outstanding questions on *what* such areas code for (see Taylor et al., 2013, for the suggestion of an overarching framework).

A third important note concerns the distinction between semanticity and lexicality—that is, carrying a meaning versus being attested as a free-standing word. Naturally, the two aspects tend to coincide in the language; existing words (e.g., “salt”) differ from pseudowords (e.g., “falt”) both because words carry meaning and they are independent chunks of letters that serve as functional units in the language. As a result, comparing words and nonwords does not allow to definitely adjudicate between the prevalence of semantic and lexical effects. Teasing the two apart, however, is absolutely critical for a theoretical understanding of reading and may shed light on the specific function of the mid-fusiform gyrus (referred to by many as the “visual word form area,” e.g., Dehaene & Cohen, 2011, a portion of the visual system whose involvement in linguistic processing has long been in the limelight of reading research). If cortical patches in these areas code for meaning, then specific linguistic functions are already performed at these early cortical stages. If, instead, this brain region is sensitive to lexicality, but not to semanticity per se, then the computations performed at this point of the visual hierarchy might be supporting linguistic and other, more general, visual material alike. That is, these areas might not feature language-specificity (e.g., Hillis et al., 2005; Wandell, 2011).

### Current Study

In the present work, we tackle these issues by taking advantage of a paradigm that recently became a prominent tool to address selective neural representations, namely, fast periodic visual stimulation (FPVS; Rossion, 2014). By relying on frequency tagging, FPVS allows to isolate the brain’s oscillatory response induced by the rapid, periodic presentation of visual items at a fixed rate (Norcia et al., 2015). The elicitation of such oscillatory responses has been recently combined with oddball designs: The regular embedding of items of a given kind (e.g., faces) in a stream of stimuli belonging to a different category (e.g., objects) creates a secondary, slower periodicity that is specific to the stimuli of interest, and that, crucially, can be captured only if the brain is indeed sensitive to the distinction (i.e., the two types of stimuli are supported by distinct neural representations). Critically, neural discrimination responses are elicited within a few minutes of stimulation, are clearly quantifiable at the predefined frequency of interest, and yield high signal-to-noise ratios. Importantly, any response is gathered implicitly, in the absence of task-induced confounds, and is selective to the dimension that differentiates the two classes of items presented within the stimulation sequence.

This approach allowed Lochy et al. (2015) to obtain selective neural responses for lexical items. Skilled readers were presented with periodic streams of stimuli (e.g., pseudowords, presented with a frequency of 10 Hz) in which word items appeared at regular intervals (every 5 items, hence with a 2 Hz frequency). Known words elicited a neural entrainment, as evidenced by a sharp 2 Hz response measured at scalp, reflecting the brain’s ability to implicitly and rapidly discriminate lexical items in a stream of readable, ortho-phonotactically legal, but nonexisting pseudowords. A series of studies adopting linguistic material (e.g., Lochy et al., 2015; Lochy et al., 2016; Lochy et al., 2018) corroborated the role of FPVS as a powerful window into the neural basis of visual word processing. Carefully controlled FPVS sequences could not only address the existence of lexical representations, but also probe the specific contributions of all the features that characterize linguistic information. Written input is indeed composed of a series of nested levels, with phonological, orthographic, and semantic factors permeating different units of processing. In the eyes of a skilled reader, known words are highly familiar, complex visual objects, which become perceptually salient after a lifetime exposure (a mechanism akin to perceptual learning; Fahle & Poggio, 2002; Gilbert et al., 2001; Goldstone, 1998; Nazir et al., 2004; Nazir & Huckauf, 2007). Structurally, visual words are combinations of known symbols (e.g., letters) that reflect the statistical co-occurrence regularities of the written language (Araújo et al., 2015; Lin et al., 2011; Rudell & Hu, 2000). Words have also consistent phonological (Aparicio et al., 2007; Braun et al., 2015; Dietz et al., 2005), morphological (Leminen et al., 2013; Leminen et al., 2016; Leminen et al., 2019; Vigliocco et al., 2006) and semantic associations (Devereux et al., 2013; Mirman & Magnuson, 2009; Price et al., 2006).

The present study aimed at investigating the contribution of each of these features by coupling magnetoencephalography (MEG) recordings with FPVS sequences, in a tightly controlled hierarchy of contrasts. The use of MEG is an important novelty as compared to most of the previous FPVS literature (but see, e.g., Pescuma et al., 2022); in fact, this technique allows a reasonable spatial resolution, while maintaining the fine temporal resolution that is required to study neural entrainment. By projecting the relevant FPVS response at source level, it is possible not only to probe much more refined linguistic and visual representations, but also to appreciate their spatial arrangement in the ventral stream and, more generally, within the language processing network. Each contrast under study tackled a specific linguistic feature, seeking evidence for its relevance in skilled reading. Specifically, we assessed the presence of an alphabetic response, by contrasting strings of consonants (e.g., “sfcl”) with sequences of items composed of artificial characters matching Roman letters on low-level visual features (BACS-2 characters; Vidal et al., 2017). The same strings of consonants were then pitted against readable, but nonexisting strings (e.g., “amsi”), to test the relevance of readability. Readable items were subsequently compared to meaningless word endings that are highly frequent in the written language (e.g., “enso”), to investigate the impact of familiarity. Such frequent units were then set against suffixes, which are frequent and meaningful sublexical items (e.g., “eria”), so that we could isolate sensitivity to meaning. The final comparison involved suffixes on one hand, and words (e.g., “idea”) on the other: A successful discrimination, here, would represent a purely lexical response, in that both classes of items are frequently attested in the written environment and consistently associated with meaning, and are exclusively differentiated on the basis of their lexical status.

## MATERIALS AND METHODS

### Participants

Twenty-one volunteers (10 females; age: *M* = 27.2, *SD* = 5.35) took part in the experiment after giving written informed consent. All participants were right-handed native Italian speakers with normal or corrected-to-normal vision and no history of linguistic or neurological impairment. The experiment was conducted in accordance with the Declaration of Helsinki and was approved by the local ethical committee of the University of Trento (Prot. 2017-020).

### Materials

**Figure 1.**
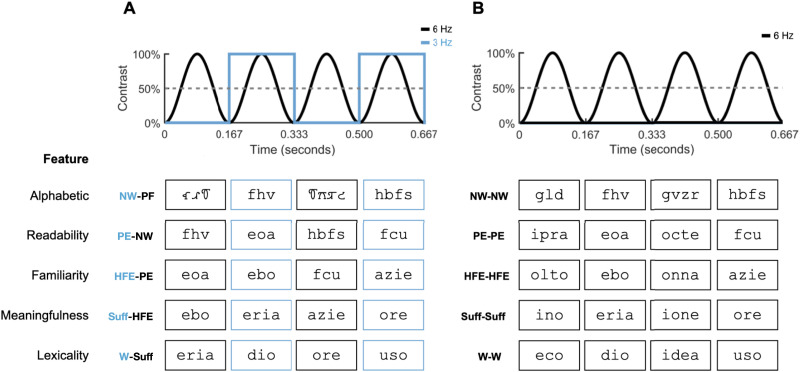
Experimental paradigm. All stimuli were presented by sinusoidal contrast modulation; the figure displays four cycles of 166.66 ms. (A) In the experimental trials (panel A) stimuli from two classes were alternated, to isolate neural responses selective to discriminative properties (e.g., letter strings and pseudo-characters differ solely on the basis of their alphabetic nature). Examples are given for the five different properties of interest: alphabetic (NW vs. PF), readability (PE vs. NW), familiarity (HFE vs. PE), meaningfulness (HFE vs. Suff), and lexicality (W vs. Suff). (B) The control trials comprised items belonging to the same category (e.g., a single stream of letter strings) and served as a baseline condition for the discriminative nature of the neural responses yielded by the experimental trials. Particularly, the difference between the 3 Hz response in experimental and baseline trials would represent a genuine discrimination between items belonging to two different experimental categories, thus reflecting the feature under study. NW = nonword, PF = pseudofont, PE = pseudoending, HFE = high frequency ending, Suff = suffix.

Stimuli (illustrated in Figure 1) comprised six categories of 32 items, all three to four elements long (*M* = 3.53, *SD* = 0.507). Words (W; e.g., “idea”) were Italian nouns, and suffixes (Suff; e.g., “eria,” in English “ery”) were derivational morphemes. Frequent endings (HFE; e.g., “enso,” akin to the English “kle”) were highly frequent, meaningless word endings attested in Italian. Pseudoendings (PE; e.g., “amsi”) were pronounceable letter strings with a regular consonant-vowel (CV) structure (i.e., fourteen items: CCV, seventeen items: VCCV, one item: VVV) that could potentially constitute ortho-phonotactically legal word endings in Italian, but are not attested in the language. Nonword stimuli (NW; e.g., “sfcl”) comprised random consonant strings and were thus unpronounceable. Finally, pseudofont (PF) strings were obtained by rendering random combinations of characters from the BACS-2 serif artificial script (Vidal et al., 2017), and resulted in strings of symbols closely matching the visual characteristics of Latin characters (i.e., number of strokes, junctions, terminations, and serifs).

Among the linguistic items, words, suffixes and frequent endings had a high and comparable written token frequency, measured by taking into account the cluster position within the word (W: *M* = 4.507, *SD* = 0.580; Suff: *M* = 4.983, *SD* = 0.533; HFE: *M* = 4.229, *SD* = 0.925), while pseudoendings and nonwords had a zero frequency, as per their definition. Frequency metrics are computed on SUBTLEX-IT (Amenta et al., 2024), which is based on a sample of 128 million words from movie subtitles (number of types = 517,564).

Given the strict constraints on the stimuli (e.g., the set of 32 suffixes we employed here is very close to the complete set of three- and four-letter Italian suffixes), the final selection includes some suboptimal items. Specifically, seven suffixes were homographic to words (e.g., “-ione,” with the Italian word for “ion”). We minimized the potential impact of these items by fully randomizing the item order for each participant; given the small number of the word-homographic suffixes, this drastically reduced (eliminated, effectively) the possibility that they might yield extra frequencies of resonance, or hamper the entrainment to the relevant frequencies by design.

### Procedure

Stimuli were presented via sinusoidal contrast modulation at a frequency of 6 Hz for 26.7 s, with each stimulus cycle lasting a total of 166.66 ms. Two types of stimulation sequences were presented for each condition. In experimental trials, the stimulation alternated between stimuli that belonged to two different categories (i.e., XYXYXY), such that stimuli from each category were presented every 333.33 ms (i.e., at a frequency rate of 6 Hz/2 = 3 Hz). In baseline trials, a sequence comprised stimuli that belonged only to one category (i.e., xxxxxx). This design ensured that in each experimental condition a differential signal (i.e., difference between alternating and baseline sequences) at the stimulation frequency of 3 Hz reflected a neural response that was selective to the property that distinguished the two categories of stimuli. It is important to appreciate that this paradigm is intrinsically nondirectional: A difference between the experimental condition with suffixes and words and the baseline condition with words only implies that the brain is sensitive to lexicality—not that it is specifically sensitive to words per se, or to suffixes per se. Stated differently, the choice of baseline (Word–Word, or Suffix–Suffix) does not affect the Word–Suffix condition. For a schematic illustration of the experimental design and examples of stimulation sequences, see Figure 1.

Five experimental conditions were used to isolate neural responses to stimuli that are alphabetic (Nonwords vs. Pseudofonts; baseline: Nonwords), readable (Pseudoendings vs. Nonwords; baseline: Pseudoendings), sublexical orthographic units (Frequent Endings vs. Pseudoendings; baseline: Frequent Endings), sublexical meaningful units (Suffixes vs. Frequent Endings; baseline: Suffixes), and lexical units (Words vs. Suffixes; baseline: Words). There were six trials per experimental condition and type of sequence, yielding a total stimulation time of 45 min: 26.7 s (trial duration) × 5 experimental conditions × 2 types of sequences (alternating, baseline) × 6 trials. Trials consisted of unique sequences of 160 stimuli, each of which was presented exactly five times in a pseudo-randomized fashion to avoid close repetitions; additionally, the order of specific items was randomized for each participant, to avoid potential list effects. The order of trial presentation was also pseudo-randomized to avoid close repetitions of specific classes of items during the course of the experimental session. Trial presentations were separated by 15 s breaks.

Participants were seated at approximately 1 m from a PROPixx DLP projector (VPixx Technologies, Canada). The screen had a 1,440 × 1,080 pixels resolution and a refresh rate of 120 Hz. Stimulus display was administered by PsychToolbox-3 (Brainard, 1997) on MATLAB R2015a (MathWorks, 2015) in a Windows environment. All stimuli were presented at the center of the screen. All alphabetic stimuli (i.e., Words, Suffixes, Frequent Endings, Pseudoendings, and Nonwords) were presented in lowercase characters, using the fixed-width Courier New font, whereas Pseudofonts were presented in BACS-2 serif font. Both fonts were emboldened by 70% from their original character weight to improve visibility. Each stimulus subtended horizontal and vertical visual angles of 2.58 and 0.64 degrees.

To ensure participants maintained a constant level of attention, they were instructed to monitor the color change of a cross presented continuously at the center of the screen. The change, from blue to red and vice versa, occurred three times in each trial, independently of the experimental manipulation. Overall, participants’ performance in the color-change detection task was close to ceiling in accuracy (*M* = 97.8%, *SD* = 14), and featured fast reaction times, (*M* = 465 ms, *SD* = 177). Moreover, it was comparable across experimental trials (NW in PF = 97%, *SD* = 16, reaction time: *M* = 460 ms, *SD* = 157; PE in NW = 97.3%, *SD* = 16, reaction time: *M* = 469 ms, *SD* = 188; HFE in PE = 98.4%, *SD* = 12, reaction time: *M* = 468 ms, *SD* = 175; Suff in HFE = 97.8%, *SD* = 14, reaction time: *M* = 476 ms, *SD* = 202; W in Suff = 98.6%, *SD* = 11, reaction time: *M* = 452, *SD* = 145) as well as baseline trials (NW in NW = 97.3%, *SD* = 16, reaction time: *M* = 466 ms, *SD* = 189; PE in PE = 98.1%, *SD* = 13, reaction time: *M* = 455 ms, *SD* = 149; HFE in HFE = 97.8%, *SD* = 14, reaction time: *M* = 477 ms, *SD* = 216; Suff in Suff = 97.6%, *SD* = 15, reaction time: *M* = 461 ms, *SD* = 160; W in W = 98.4%, *SD* = 12, reaction time: *M* = 462 ms, *SD* = 173). A one-way analysis of variance revealed no statistically significant differences across conditions (accuracy: *F*(9, 1,230) = 0.522, *p* = 0.859; reaction time: *F*(9, 1,230) = 0.894, *p* = 0.53).

### MEG Acquisition, Preprocessing, and Frequency Analysis

MEG data were recorded using a whole-head 306 sensor (204 planar gradiometers; 102 magneto-meters) Vector-view system (Elekta Neuromag, Helsinki, Finland). Participants’ head position was continuously determined with respect to the MEG helmet through five head position indicator coils (HPIs). MEG signals were recorded at a sampling rate of 1000 Hz and online band-pass filtered between 0.1 and 300 Hz. At the beginning of each experimental session, fiducial points of the head (the nasion and the left and right pre-auricular points) and a minimum of 300 other head-shape samples were digitized using a Polhemus FASTRAK 3D 519 digitizer (Fastrak Polhemus, Inc., Colchester, VA, USA). Raw data were processed through MaxFilter 2.0 (Elekta Neuromag). For each participant, bad channels were identified via visual inspection, and interpolated. Head displacements were inspected and corrected through realignment to a single reference. After applying movement compensation, external sources of noise were separated and removed by applying the temporal extension of signal space separation (tSSS; Medvedovsky et al., 2009; Taulu & Hari, 2009; Taulu & Simola, 2006).

Preprocessing and analysis were performed in MATLAB with a combination of Fieldtrip (Oostenveld et al., 2011), Brainstorm (Tadel et al., 2011), and custom scripts. Continuous recordings from each participant were band-pass filtered (0.1–100 Hz), downsampled (250 Hz) and epoched into 26.7 s trials, which were realigned to the onset of the first stimulus (via a photodiode). Segments contaminated by artifacts were identified through visual inspection and manually removed (1.11%). To remove eye movements and heartbeat related artifacts from the MEG signal we performed an independent component analysis (ICA; Jutten & Herault, 1991), separately for magnetometers and planar gradiometers. Eye movement and pulse-related components were captured by correlating the independent component (IC) time series with that of electrooculography and electrocardiography channels.

For each participant, trials within each condition were averaged, and submitted to a fast Fourier transformation. Given the length of the epochs, the frequency resolution was 1/26.7 = 0.0374 Hz. The spectra were then baseline-corrected by subtracting from each frequency bin the mean of the surrounding 20 bins (10 from each side, excluding local minima, maxima, and immediately adjacent bins, as in, e.g., Dzhelyova & Rossion, 2014); the response of interest was then defined as the baseline-corrected amplitude at 3 Hz. A significant discrimination response, indexing neural entrainments elicited by items belonging to different categories, was assessed by comparing the 3 Hz response in each experimental condition (e.g., Words in Suffixes) with the corresponding baseline (i.e., Words in Words).

#### Sensor space analysis

Sensor level analyses were run at whole-brain level through a non-parametric cluster permutation test (Maris & Oostenveld, 2007). Differences at 3 Hz between the experimental and the baseline conditions were assessed separately for magnetometers and combined planar gradiometers, by considering a minimum neighborhood distance of 6 mm between sensors. Statistical significance was assessed through a one-tail, dependent sample *t* test with Monte Carlo estimates over 5,000 permutations (significance level: *p* < 0.05).

#### Source space analysis

Distributed minimum-norm source estimation (MNE; Hämäläinen & Ilmoniemi, 1994) was applied following the standard procedure in Brainstorm (Tadel et al., 2011). For 20 participants, anatomical T1-weighted magnetic resonance images were acquired during a separate session in a Prisma 3 T scanner (Siemens, Erlangen, Germany) using a 3D MPRAGE sequence, 1 mm^3^ resolution, TR = 2,140 ms, TI = 900 ms, TE = 2.9 ms, flip angle 12°, and segmented in Freesurfer (Fischl, 2012). Co-registration of MEG sensor configuration and the reconstructed scalp surfaces was based on around 300 scalp surface locations. As one participant did not undergo MRI acquisition, we warped the default anatomy to match the shape defined by the digitized points. Individual noise covariance matrices were computed from 1 s prestimulus interval in all the available trials for each participant. The forward model was obtained using the overlapping spheres method (Huang et al., 1999) as implemented in Brainstorm. Fourier-transformed regression coefficients were then projected onto a 15,000 vertices boundary element using a dynamic statistical parametric mapping approach (dSPM; Dale et al., 2000), assuming dipole sources to be perpendicular to the cortical surface. Individual results were spatially smoothed (3 mm FWHM) and projected to a default template (ICBM152).

Differences at 3 Hz between experimental and baseline conditions were then assessed at source level in the vertices of predefined regions of interest (ROIs), obtained from the Desikan-Killiany cortical atlas (Desikan et al., 2006). The ROIs selected for the source-level analysis corresponded to some of the most prominent cortices involved in reading, such as fusiform (e.g., Dehaene et al., 2002), lingual (e.g., Raschle et al., 2011), inferior parietal (e.g., Sliwinska et al., 2015), inferior temporal (e.g., Dien et al., 2013), lateral occipital (e.g., Borowsky et al., 2007), and middle temporal (e.g., Turkeltaub et al., 2003). On each ROI, significant responses were assessed via nonparametric cluster permutation test (*N* = 5,000, *p* < 0.05; Maris & Oostenveld, 2007). Nonsignificant effects were here explored through JZS Bayes Factor analysis (BF_10_, scale factor *r* = 0.707; Rouder et al., 2009), which provides quantifiable evidence in support of H1 or H0, thus allowing to support the null hypothesis itself (Leppink et al., 2017).

## RESULTS

### Sensor Space

**Figure 2.**
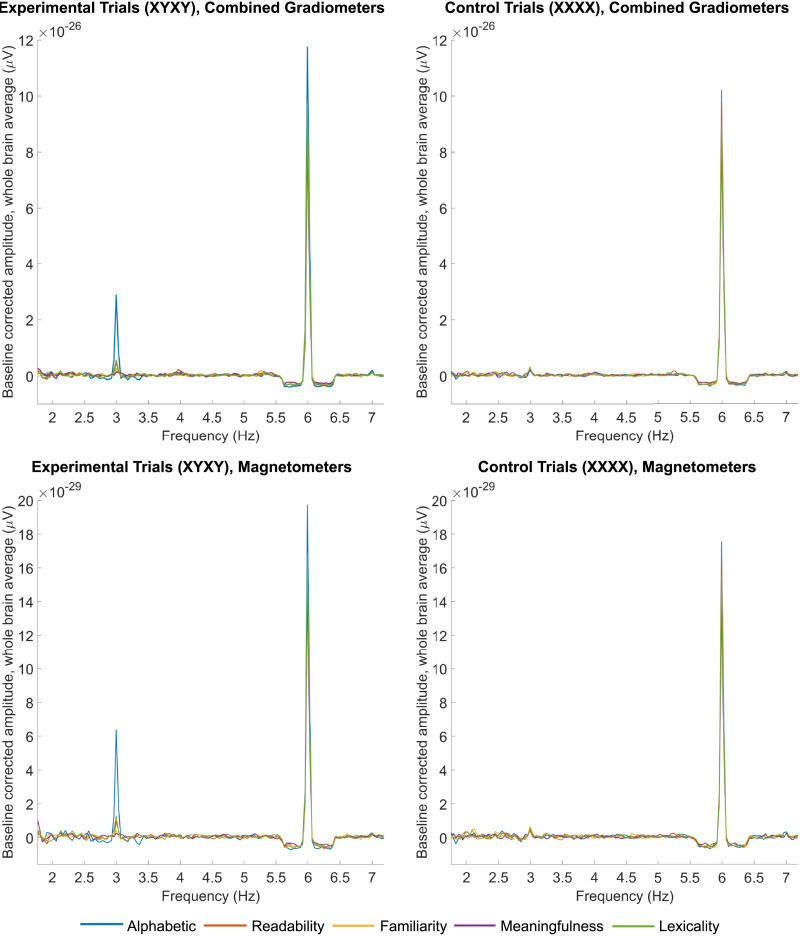
Grand-averaged, whole-brain baseline-corrected amplitude (V) spectra for the different conditions across sensor types. Critically, the profiles indicate that the response is confined to the frequencies of interest (3 and 6 Hz).

**Figure 3.**
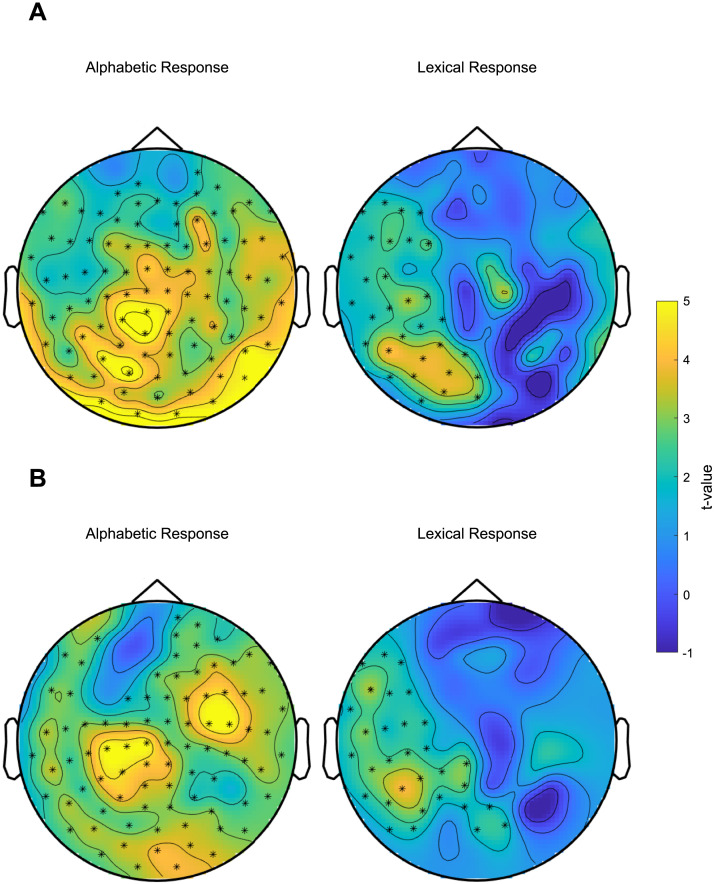
Sensor level results. One significant, largely diffused cluster indicated the discrimination of alphabetic stimuli, while a left-lateralized significant cluster was associated with lexical discrimination. The topography of the effects is comparable across planar gradiometers (A) and magnetometers (B) for both discrimination responses.

The profile of the neural responses gauged at the frequencies of interest (3 and 6 Hz) for both experimental and control trials is depicted in Figure 2. As shown in Figure 3, a clear discrimination response indicated sensitivity to the alphabetic nature of the items (NW–PF vs. NW–NW), emerging in a diffused area for both planar gradiometers (*t*_(20)_ = 311.13, *p* = 0.0002, *g* = 0.83 [0.53, 1.13]) and magnetometers (*t*_(20)_ = 274.17, *p* = 0.0002, *g* = 0.81 [0.49, 1.12]). Lexical items embedded in suffixes (W–Suff vs. W–W) also elicited a marked discrimination response, with left-lateralized topography for both planar gradiometers (*t*_(20)_ = 80.30, *p* = 0.0004, *g* = 0.65 [0.12, 1.16]) and magnetometers (*t*_(20)_ = 74.28, *p* = 0.0002, *g* = 0.71 [0.18, 1.22]).

No discrimination response emerged as statistically significant for the other contrasts. Readability (PE–NW vs. PE–PE) resulted in a cluster that did not reach significance in planar gradiometers (*t*_(20)_ = 9.39, *p* = 0.13, *g* = 0.61 [–0.91, 2.01]) and no cluster for magnetometers. Familiarity (HFE–PE vs. HFE–HFE) produced a nonsignificant cluster for magnetometers (*t*_(20)_ = 8.81, *p* = 0.233, *g* = 1.03) and no cluster for gradiometers. No significant cluster emerged for meaningful sublexical units (Suff–HFE vs. Suff–Suff).

### Source Space

**Figure 4.**
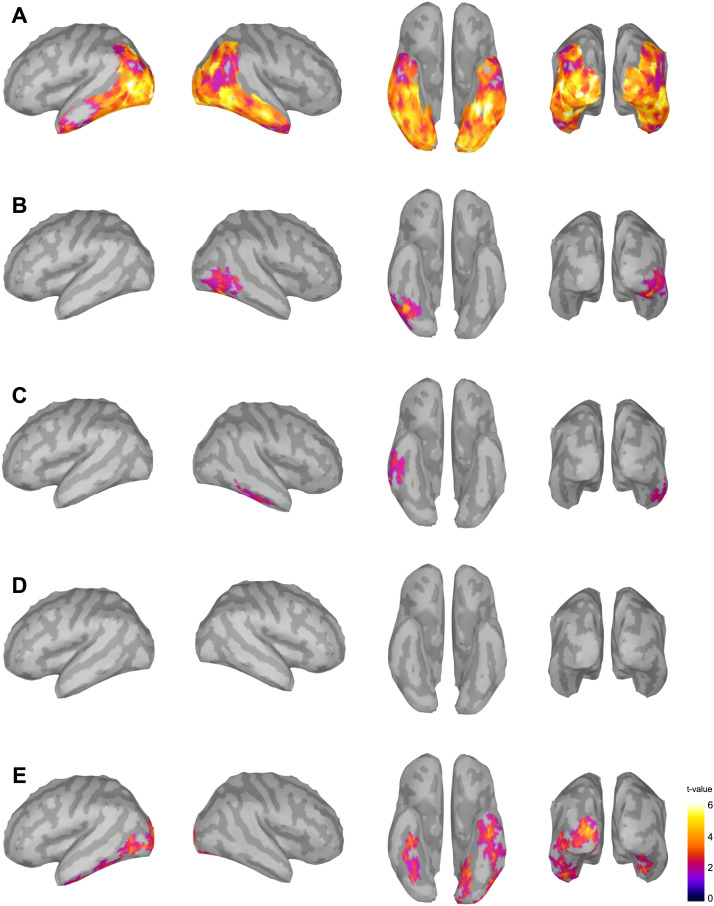
Source level results. Significant discrimination responses (*p* < 0.05) for the five linguistic properties under study: (A) Alphabetic, (B) Readability, (C) Familiarity, (D) Meaningfulness, (E) Lexicality), displayed (from left to right) with left, right, ventral, and posterior views.

Significant discrimination responses in the predefined ROIs are displayed in Figure 4. At source level, a 3 Hz discrimination response for the alphabetic nature of the stimuli (NW–PF vs. NW–NW) emerged bilaterally in all the areas of interest (as summarized in Table 1).

**Table 1.** Alphabetic response (PF–NW vs. NW–NW) source level results.

ROI	*t* value	Effect size *g*	Pr(>|*t*|)	
Left fusiform	1059.21	1.57 [1.36, 1.77]	<0.001	***
Right fusiform	956.08	1.91 [1.69, 2.12]	<0.001	***
Left inferiorparietal	1060.81	1.35 [1.18, 1.52]	<0.001	***
Right inferiorparietal	1315.81	1.37 [1.22, 1.52]	<0.001	***
Left inferotemporal	903.83	1.29 [1.10, 1.48]	<0.001	***
Right inferotemporal	1052.57	1.57 [1.38, 1.75]	<0.001	***
Left lateral occipital	1609.02	1.48 [1.32, 1.65]	<0.001	***
Right lateral occipital	1313.58	1.62 [1.45, 1.79]	<0.001	***
Left lingual	921.25	1.59 [1.37, 1.80]	<0.001	***
Right lingual	729.36	1.70 [1.47, 1.92]	<0.001	***
Left middle temporal	603.84	1.03 [0.82, 1.25]	<0.001	***
Right middle temporal	1141.24	1.38 [1.20, 1.55]	<0.001	***

*Note*. PF = pseudofont, NW = nonword.

A discrimination between readable stimuli and strings of consonants (PE–NW vs. PE–PE) yielded a right-lateralized profile, involving fusiform (*t*_(20)_ = 161.31, *p* = 0.0216, *g* = 1.23 [0.82, 1.63]), inferotemporal (*t*_(20)_ = 134.31, *p* = 0.0206, *g* = 1.30 [0.86, 1.72]), lateral occipital (*t*_(20)_ = 204.39, *p* = 0.024, *g* = 1.16 [0.82, 1.49]), and middle temporal cortices (*t*_(20)_ = 105.35, *p* = 0.0332, *g* = 0.92 [0.48, 1.34]).

Familiarity (HFE–PE vs. HFE–HFE) elicited significant responses only in the right inferior temporal area (*t*_(20)_ = 220.03, *p* = 0.0178, *g* = 1.14 [0.81, 1.47]).

Consistent with the results observed in sensor space, suffixes embedded in frequent endings yielded no significant response in the predefined ROIs. This null result was further explored through a JZS Bayes factor analysis across ROIs, which, where conclusive, provided moderate evidence in favor of the null hypothesis (as described in Table 2).

**Table 2.** Suff–HFE vs. Suff–Suff contrast source level Bayes factor analysis.

ROI	No. of vertices	BF_10_ < 1/3	BF_10_ > 3
Left fusiform	268	156 (58%)	0
Right fusiform	255	186 (72%)	0
Left inferiorparietal	351	70 (19%)	39 (11%)
Right inferiorparietal	421	219 (52%)	20 (5%)
Left inferotemporal	307	155 (50%)	0
Right inferotemporal	316	213 (67%)	9 (3%)
Left lateral occipital	371	207 (55%)	4 (1%)
Right lateral occipital	367	251 (68%)	0
Left lingual	246	154 (62%)	0
Right lingual	227	186 (82%)	0
Left middle temporal	277	191 (69%)	9 (3%)
Right middle temporal	324	205 (63%)	19 (6%)

*Note*. For each region of interest, the table reports the total number of vertices, the ones providing moderate evidence in favor of the null hypothesis (i.e., with BF_10_ < 1/3), and those moderately supporting the alternative (i.e., with BF_10_ > 3). Suff = suffix, HSE = high frequency endings.

Differences between words and suffixes (W–Suff vs. W–W) were traced in bilateral fusiform (left: *t*_(20)_ = 227.12, *p* = 0.0174, *g* = 1.11 [0.77, 1.44]; right: *t*_(20)_ = 283.35, *p* = 0.004, *g* = 0.94 [0.65, 1.22]), together with two significant clusters in left inferior temporal (first: *t*_(20)_ = 230.97, *p* = 0.0206, *g* = 1.01 [0.68, 1.33]; second: *t*_(20)_ = 186.03, *p* = 0.029, *g* = 0.93 [0.55, 1.29]), left lateral occipital (*t*_(20)_ = 580.00, *p* = 0.0008, *g* = 0.77 [0.57, 0.97]), and left lingual (*t*_(20)_ = 436.91, *p* = 0.004, *g* = 1.23 [0.98, 1.48]).

## GENERAL DISCUSSION

The present study investigated the neural underpinnings of visual word identification, by asking which linguistic features might be rapidly and automatically discriminated by the reading brain. With this aim, we paired MEG recordings with FPVS (Rossion, 2014) constructed specifically to isolate some of the fundamental features of written text, in a carefully controlled hierarchy of nested contrasts. In this design, observing neural entertainment at the frequency with which classes of items were presented implicitly indexes a selective discrimination for the feature that differentiates the two groups of items. Interleaving words (e.g., “idea”) and suffixes (e.g., “eria”) revealed a strong lexicality response, already detectable at sensor level. Similarly, the alternation of pseudo-characters and letter strings (e.g., “sfcl”) revealed a strong sensitivity to the alphabetic nature of the stimuli, also observable at both sensor and source levels. More subtle contrasts, addressing the role of readability (with strings of consonants, e.g., “sfcl” vs. pseudoendings, e.g., “ampi”) and familiarity of letter strings (with high frequency endings, e.g., “enso” vs. pseudoendings, e.g., “ampi”), were captured only at source level. Notably, our results did not reveal a meaningfulness response, as assessed by contrasting suffixes (e.g., “eria”) with equally frequent, but meaningless word endings (i.e., “enso”).

One way to interpret these findings is that the reading system is automatically responsive to lexicality. Such a response is characterized by a predominantly left-lateralized profile, arising in areas typically associated with word identification and processing, such as left lateral occipital, lingual and inferotemporal cortices (Binder & Price, 2001; Borowsky et al., 2007), as well as the bilateral fusiform gyrus (Cohen et al., 2002; Dehaene et al., 2002; Fiez & Petersen, 1998; Puce et al., 1996). This selective neural discrimination for lexicality lends some support to the existence of an orthographic lexicon, consistently with more recent neural models of reading (Taylor et al., 2013). Nevertheless, the spatial resolution of noninvasive human neuroimaging in general, and of MEG in particular, requires a word of caution in interpreting these results, especially at a cognitive level. Specifically, our findings cannot pinpoint whether this lexical response originates from a set of individual neurons specifically selective to words per se, or rather from a coordinated, large ensemble of neurons with a less granular preference. Therefore, the present study cannot arbitrate between localist and distributed accounts of lexical neural codes at a mechanistic level (Bowers, 2009, 2017; Quian Quiroga & Kreiman, 2010; Roy, 2012; Vankov & Bowers, 2017).

Neuroimaging evidence has been fairly elusive as to whether the reading brain responds specifically to existing words (as opposed to well formed strings of letters, e.g., Binder et al., 2006; Dehaene et al., 2005; Pammer et al., 2004; Price et al., 1996; Wydell et al., 2003). The adoption of neural adaptation techniques has provided a decisive methodological boost in this direction, by allowing the tapping into selective neuronal tunings to stimulus features (Grill-Spector et al., 2006; Norcia et al., 2015). Importantly, however, while previous studies succeeded in capturing selective adaptation to lexical forms (e.g., Glezer et al., 2009; Lochy et al., 2015), they generally did so by pitting words against pseudowords, thus adopting two classes of items that differ in more than one relevant dimension. Written words are indeed meaningful linguistic objects, with a known phonological and orthographic form, while well-structured pseudowords are, albeit pronounceable (Taylor et al., 2013), unknown strings of letters. Contrariwise, the lexical response obtained in the present study stems from an unprecedentedly tight comparison, realized by contrasting fully fledged, real words with suffixes. Morphemes like “-ness” or “-er” are attested in the language with high frequency, and due to their derivational properties, they alter the meaning of stems they are combined with in a highly predictable and consistent manner (e.g., kind*ness*, high*ness*, sing*er*, danc*er*)—thus, they have a specific meaning (Bloomfield, 1933; Bybee, 1988). Naturally, the nature of this meaning is often different from words; for example, suffixes might be seen as carrying functional information, which modifies the core lexical message carried by the stem (e.g., “X-ness” as the state of being X; Marelli & Baroni, 2015—although this does not have implications of strength, or relative importance; for example, suffixes are generally assumed to be the morphological head of derived words).

Similar to words, however, suffixes forge strong and consistent associations between their orthographic form and a semantic concept (e.g., the suffix “-er” conveys agency), and they mostly differ from words in their sublexical, rather than lexical, status, in that they cannot appear in isolation as independent linguistic units. Therefore, the adoption of morphemes allows to uniquely overcome the rather coarse characterization of the lexical discrimination obtained with pseudowords, and supports the presence of a neural response that is specifically lexical, not related to meaning, frequency of occurrence, or readability alone.

Indubitably, sublexical morphemes such as suffixes play a fundamental function in visual word identification. Behavioral evidence has extensively supported the role of morphemes in the recognition of complex words (e.g., Amenta & Crepaldi, 2012; Bonandrini et al., 2023; Giraudo & Voga, 2014; Rastle & Davis, 2008), including experiments where, similar to the present study, suffixes where presented in isolation and under tight visual conditions (masked priming; e.g., Andoni Duñabeitia et al., 2008). This was further corroborated by several neuroimaging studies (e.g., Beyersmann et al., 2021; Davis et al., 2004; Devlin et al., 2004; Gold & Rastle, 2007; Lavric et al., 2012; Lehtonen et al., 2011; Lewis et al., 2011; Pescuma et al., 2022; for a recent review, see Leminen et al., 2019). Nevertheless, the vast majority of the available studies investigated the role of morphemes by embedding them in a lexical context (i.e., morphologically complex words, e.g., “kind-*ness*,” or pseudowords, e.g., “table-*ness*”), and thus leaves their specific neural characterization somewhat underspecified. The present study provides novel insight by indicating that, when presented in isolation, suffixes are not reliably distinguished from frequent word endings. Such a finding nicely reckons with recent experimental evidence obtained in artificial lexicon studies showing that skilled readers can carve affix-like units on the sole basis of their frequency of occurrence, and even in the absence of phonological or semantic information (e.g., Chetail, 2017; Lelonkiewicz et al., 2020, 2023). Collectively, this body of evidence does not abide by cognitive models of morphological processing that assume dedicated representations for meaningful, sublexical units (e.g., Crepaldi et al., 2010; Taft, 2004; Taft & Nguyen-Hoan, 2010) and is better aligned with accounts emphasizing perceptual and orthographic mechanisms for the decomposition of complex words (e.g., Grainger & Beyersmann, 2017). Particularly, Grainger and Beyersmann (2017) theorize that while the recognition of sublexical units is achieved on the basis of orthographic factors, their semantic activation is primarily driven by the lexical context in which they appear (e.g., the meaning of “-er” would be activated when the suffix is presented in an adequate context, like “sing-*er*”). Coherently, and in spite of their morphological status, isolated suffixes would be no more perceptually salient than other highly frequent word endings.

Taken together, our results seem to reflect the sensitivity of the reading system to form-based regularities, by tapping into the bottom-up processing of visuolinguistic material. Words stood out as independent units even if compared with another set of meaning-bearing items, consistently with theories of perceptual learning (Fahle & Poggio, 2002; Gilbert et al., 2001; Goldstone, 1998). The lexical knowledge available to skilled readers is indeed reliant not only on linguistic information, but also on the visual familiarity that results from an extensive experience with written text, where frequent and repeated encounters with printed words would consolidate their representation as complex but unitary shapes, rather than combination of features (Gilbert et al., 2001; Nazir, 2000; Nazir & Huckauf, 2007). Consequently, individual words would become privileged units of processing that “pop-out” (Nazir et al., 2004) to the eyes of a skilled reader, particularly if displayed in their most prototypical form (as, e.g., in a horizontal orientation, Nazir & Huckauf, 2007; Wimmer et al., 2016). Critically, word tokens are generally surrounded by empty spaces, which provide privileged anchor points to infer letter information and, subsequently, word identity (Fischer-Baum et al., 2011; Grainger & Beyersmann, 2017; Jacobs et al., 2013). Such perceptual salience might not be comparably bestowed upon suffixes, which, although frequent and meaningful, are bound to appear within complex words and are never encountered independently. Coherently, bound morphemes like suffixes were not significantly discriminated from highly frequent, but meaningless word endings; both classes of items are comparably familiar in their form arguably and are equally supported by the perceptual experience of skilled readers. These considerations raise an interesting point about the contribution of more linguistic and more perceptual factors to our reading experience—and, more importantly, how they shape the cognitive and neural architecture underlying reading. Of course, lexicality is a very rich linguistic construct, which has important ramifications in virtually all aspects of our linguistic experience (e.g., phonology, syntax). Yet, we always see words surrounded by blanks, so it is not so unlikely that this becomes a fundamental piece of information that our lexical system captures. We conceive these perceptual and linguistic factors as integral parts of our visual word identification system; as allies, not competitors, in determining the way in which the brain processes letters and words. Certainly, the present study cannot really tease these perceptual and linguistic factors apart; this was not the goal of the present work. Future studies, perhaps capitalizing upon orthographic systems that do not build upon inter-word spacing (e.g., Mandarin Chinese), might shed some light on the respective contributions of visual (e.g., boundedness) and linguistic (i.e., meaning) factors in reading.

Additionally, the role of context in morpheme processing extends beyond perceptual factors, as attested, for instance, by studies focusing on inflectional affixes. Word and phrase contexts are critical for affix interpretation in languages with rich inflectional systems (Franzon & Zanini, 2023; Pescuma et al., 2021), as well as for disambiguating homographs (Franzon et al., 2021; Franzon & Zanini, 2023). Moreover, the presence of semantic content in inflectional morphemes can even extend as far as to affect word processing and recognition (Arcara et al., 2019; Zanini et al., 2020), suggesting that the presence of a minimal context plays a fundamental role in allowing readers to access the meaning of sublexical units.

Admittedly, this might depend, at least in part, on the specific paradigm adopted. FPVS enhances the visual, fast, automatic, and implicit processing of letters and strings; therefore, it certainly taps into what can be thought as the perceptual front-end of the reading system. This might reconcile the highly relevant role that morphemes play in visual word identification (e.g., Amenta & Crepaldi, 2012; Bonandrini et al., 2023) with the lack of a suffix-specific response observed in the present work. Nevertheless, the present data indicate that words and suffixes have different statuses in the visual word identification system, a conclusion that speaks against what many cognitive models postulate (e.g., Crepaldi et al., 2010; Grainger & Ziegler, 2011; Taft & Nguyen-Hoan, 2010).

With respect to the lack of semantic effects, it is interesting to note that some FPVS study was able to elicit meaning-based responses (Stothart et al., 2017). However, these effects emerged with images, not words, and using slower oddball cycles; these methodological differences might be critical for tackling higher-level processing. There is also neuroimaging evidence suggesting very early semantic activation for written words (e.g., Chen et al., 2015; Sulpizio et al., 2022). However, this literature did not use FPVS, and the specific timing of meaning activation in the brain after the presentation of written words is still quite inconsistent across studies (e.g., Vignali et al., 2023).

The selective neural responses for alphabetic stimuli, as well as for readability and familiarity, also sit well with a bottom-up account of the present results. Strings of consonants embedded in pseudo-characters elicited a strong and diffused response, involving all the predefined ROIs considered. Such a pervasive alphabetic response suggests that, despite pseudo-characters being carefully matched onto letters’ low-level visual features (Vidal et al., 2017), letter-based configurations were markedly more familiar to skilled readers (Lochy et al., 2015; Lochy et al., 2016; Lochy et al., 2018; Thesen et al., 2012; van de Walle de Ghelcke et al., 2020; Vinckier et al., 2007; Wang et al., 2021). When contrasted with strings of consonants, readable but non-attested sequences of letters (e.g., “ampi”) elicited a right-lateralized response encompassing fusiform, lateral occipital, and both middle and inferior temporal ROIs, areas reportedly involved in vowel processing, as opposed to consonants (Carreiras et al., 2009; Carreiras & Price, 2008) and non-speech (Obleser et al., 2006; Uppenkamp et al., 2006). Critically, ortho-phonotactically legal items are not only readable, but also more word-like (as opposed to consonant strings), a feature considered to be at the core of the neural underpinnings of reading (e.g., Binder et al., 2006; Vinckier et al., 2007), and consistent with a form-based regularity account of the present findings. In a transparent orthography like Italian, orthographic units are unambiguously associated with a phonological pattern, thus hampering a clear-cut distinction between the effects of familiarity and readability per se. Nevertheless, recent neuroimaging evidence obtained in Hebrew (Weiss et al., 2015) appears to support a privileged role for familiarity over orthographic transparency. By exposing skilled readers to words with vowel sounds rendered either through vowels alone, or with the adoption of diacritic markers, Weiss et al. (2015) observed that the more familiar format (i.e., without diacritics) provided a major processing advantage. This advantage overrode the increased transparency ensured by the presence of diacritics, hence pinpointing visual familiarity as a key feature in the neural processing of readable stimuli (see, e.g., Chetail & Boursain, 2019; Kinoshita et al., 2021; Marcet et al., 2020; Perea et al., 2020; Perea et al., 2022, for recent behavioral investigations on the topic). Critically, Italian has a much more transparent orthography than Hebrew, and this might affect readers in their propensity to rely on visual familiarity; further studies relying on direct cross-linguistic comparisons are required to better describe the roles of familiarity and orthographic depth in reading performance.

Finally, the contrast between readable but nonexisting word endings (e.g., “ampi”) and highly frequent word endings (e.g., “enso”) resulted in a selective neural entrainment sourced in an anterior portion of the right inferotemporal ROI. Behavioral research on reading pullulates with effects of written frequency, which have been often considered proxies of learned representations (Baayen et al., 2007; Burani & Thornton, 2003; Colé et al., 1989; Monsell et al., 1989; Preston, 1935; Taft, 1979, 2004; see, e.g., Brysbaert et al., 2018; Ellis, 2002, for reviews). Neuroimaging studies indicate that frequency effects can be traced throughout several visual word identification processes (Barber & Kutas, 2007), and that frequency-modulated activations might be housed in occipitotemporal regions (e.g., Frost et al., 2005; Keller et al., 2001; Kuo et al., 2003; Montani et al., 2019; Vinckier et al., 2007; but see, e.g., Fiebach et al., 2002; Fiez et al., 1999; Ischebeck et al., 2004, for diverging patterns of results). Notably, written frequency effects are reminiscent of a more general recognition mechanism of extraction and storage of recurring patterns, including words, faces, and other salient visual objects (Kronbichler et al., 2004; Vidal et al., 2021). In keeping with this conjecture, the selective neural entrainment elicited by high-frequency clusters in the present study stems from a portion of the inferior temporal cortex, which constitutes a cornerstone of visual object encoding (DiCarlo et al., 2012). Particularly, this area has been attested to support the processing of orthographic items in primates (Rajalingham et al., 2020) and qualifies as a powerful visual processing resource to be recycled (Dehaene & Cohen, 2007) by the more phylogenetically recent reading system.

The absence of a discrimination response for meaningfulness (i.e., between high-frequency endings and suffixes) and the presence of effects that predominantly relate to visual familiarity (e.g., frequent word endings vs. unattested letter strings) seem to suggest that the present data are mostly driven by bottom-up processes. This conclusion is also supported by a series of methodological considerations. The FPVS technique allows to detect automatic and implicit neural discrimination responses within a few minutes of stimulation, by capitalizing on a rapid presentation rate and absence of explicit engagement with the experimental material (Liu-Shuang et al., 2014; Norcia et al., 2015; Rossion, 2014). Critically, stimuli are presented via sinusoidal contrast modulation (from white background to full contrast and back) with a frequency of 6 Hz, thus each item remains on screen for about 167 ms, reaching full contrast at 83 s, and with an actual visibility duration of around 140 ms (considering that stimuli can be recognized at low contrast levels, such as 20%, Lochy et al., 2015, 2016). Such a brief presentation is complemented with the perceptual masking induced by sequential stimulus presentation, which unfolds without any interstimulus interval (in line with rapid serial visual presentation [RSVP] paradigms; see Retter et al., 2018, for a related discussion). As a result, the present FPVS design is likely to tap into rather early stages of processing, which are probably informed more by bottom-up, visual and orthographic information rather than by top-down, higher level information (such as semantics).

Remarkably, the demanding nature of the stimulation stream is also consistent with the spatial profile of the more subtle responses obtained along the hierarchy of contrasts. Indeed, while linguistic processes are generally associated with activity in the left hemisphere (Dehaene et al., 2001; Pinel & Dehaene, 2010), the right hemisphere is reportedly more resilient to fast and degraded visual presentations of alphanumeric stimuli (e.g., Asanowicz et al., 2017; Hellige & Michimata, 1989; Jonsson & Hellige, 1986; Michimata & Hellige, 1987; Sergent & Hellige, 1986; Verleger et al., 2011; Verleger et al., 2013). Coherently, while a selective lexical response resulted in a prototypical left-lateralized profile, more subtle, sublexical units could enjoy weaker support from pre-existent linguistic representations, which allowed the right-lateralized, perceptual response to be more easily captured. Future research is needed to address the impact of different experimental parameters, by systematically tuning the stimulation frequency to the feature of interest and assessing whether different presentation rates could further qualify the neural entrainment hereby observed. (For a related discussion, see Alonso-Prieto et al., 2013; Rossion, 2014.)

Overall, the present results contribute to our understanding of the general architecture of the visual word identification system, and contribute to addressing some critical open issues, such as the relationship between letter statistics and linguistic units, as well as the role of lexicality and its relationship with meaning. When semantics are decoupled from existence as an independent lexical unit—that is, from being a recurrent string of letters flanked by blanks—the cognitive system does not show much sensitivity to the former and quite a lot for the latter (at least as far as assessed in a FPVS setting). One hypothesis we can advance in this regard is that FPVS might reduce the importance of the usual feedback signal that the fusiform gyrus receives from higher-level language circuitry (Ben-Shachar et al., 2007; Devlin et al., 2006; Wandell, 2011), boosting visual effects and hampering the semantic factors. In such case, the widespread lexicality effect observed could be interpreted as mostly reliant on the bottom-up process of perceiving a unitary visual object: the word. The FPVS lexicality signal attested here extends well beyond the posterior fusiform, and includes the right anterior fusiform gyrus, the lateral occipital gyrus and the inferior temporal gyrus on the left. These data would then suggest that the sensitivity of visual string processing to chunks of letters that co-occur with specific statistical patterns climbs up the visual identification system much more than what might have been previously thought. Not only the posterior fusiform would be more about letter co-occurrence statistics than meaning and “proper” linguistic content (e.g., Dehaene & Cohen, 2011; Hillis & Caramazza, 1991; Nobre et al., 1994), but the statistics and, more generally, the more perceptual aspects of visual word identification would play a relevant role upstream. This would be in line with data (e.g., Vidal et al., 2021) and theories (e.g., Dehaene & Cohen, 2007) suggesting that the computational structure of the visual word identification system might be strongly determined by the pre-existing, biologically constrained processing mechanisms that reside in these areas.

In conclusion, the present study capitalized on FPVS and MEG recordings to shed some new light on which linguistic features underpin reading. Implicit discrimination responses emerged in a tightly controlled hierarchy of contrasts, whose extremes revealed a strong sensitivity to letters and lexical items. Sensitivity to the intermediate layers—the mere association with meaning, familiarity, and readability—was generally weaker, if present at all. Taken together, these results provide novel insight into the brain’s sensitivity to form-based regularities, and highlight the relevance of perceptual familiarity at the early stages of visual word identification.

## FUNDING INFORMATION

Davide Crepaldi, HORIZON EUROPE European Research Council (https://dx.doi.org/10.13039/100019180), Award ID: 679010. Davide Crepaldi, Ministero dell’Istruzione, dell’Università e della Ricerca (https://dx.doi.org/10.13039/501100003407), Award ID: 2015PCNJ5F.

## AUTHOR CONTRIBUTIONS

**Mara De Rosa**: Conceptualization: Lead; Formal analysis: Lead; Investigation: Lead; Software: Equal; Visualization: Lead; Writing – original draft: Lead; Writing – review & editing: Equal. **Lorenzo Vignali**: Data curation: Equal; Software: Equal; Visualization: Supporting; Writing – review & editing: Equal. **Anna D’Urso**: Data curation: Equal. **Maria Ktori**: Conceptualization: Equal; Methodology: Equal; Supervision: Equal; Writing – review & editing: Equal. **Roberto Bottini**: Conceptualization: Supporting; Funding acquisition: Equal; Writing – review & editing: Equal. **Davide Crepaldi**: Conceptualization: Supporting; Funding acquisition: Equal; Writing – original draft: Equal.

## CODE AND DATA AVAILABILITY STATEMENT

Data and materials for the experiment reported in this study are available at https://osf.io/u58w7/.

## TECHNICAL TERMS

Skilled reader: An individual proficient in decoding, processing, and understanding written language.
Morphemes: The smallest units in language that carry meaning or grammatical function.
Orthographic system: The neural network responsible for recognizing and processing written language.
Pseudowords: Plausible strings of letters or sounds that follow the rules of a language but do not correspond to meaningful words.
Nonwords: Implausible combinations of letters or sounds that do not comply with the rules of a given language.
Frequency tagging: Involves presenting stimuli at specific frequencies to measure (potential) brain responses occurring at those same frequencies.
Magnetoencephalography (MEG): Measures brain activity using magnetic fields; helps study neural processes with high precision.
